# Müller matrix polarimetry for pancreatic tissue characterization

**DOI:** 10.1038/s41598-023-43195-7

**Published:** 2023-09-29

**Authors:** Paulo Sampaio, Maria Lopez-Antuña, Federico Storni, Jonatan Wicht, Greta Sökeland, Martin Wartenberg, Pablo Márquez-Neila, Daniel Candinas, Brice-Olivier Demory, Aurel Perren, Raphael Sznitman

**Affiliations:** 1 ARTORG Center, University of Bern, Bern, Switzerland; 2 Department of Visceral surgery and medicine, Bern University Hospital, Bern, Switzerland; 3 Center for Space and Habitability, University of Bern, Bern, Switzerland; 4 Institute of Tissue Medicine and Pathology, University of Bern, Bern, Switzerland

**Keywords:** Image processing, Machine learning, Pancreatic disease, Translational research, Imaging and sensing, Polarization microscopy

## Abstract

Polarimetry is an optical characterization technique capable of analyzing the polarization state of light reflected by materials and biological samples. In this study, we investigate the potential of Müller matrix polarimetry (MMP) to analyze fresh pancreatic tissue samples. Due to its highly heterogeneous appearance, pancreatic tissue type differentiation is a complex task. Furthermore, its challenging location in the body makes creating direct imaging difficult. However, accurate and reliable methods for diagnosing pancreatic diseases are critical for improving patient outcomes. To this end, we measured the Müller matrices of ex-vivo unfixed human pancreatic tissue and leverage the feature-learning capabilities of a machine-learning model to derive an optimized data representation that minimizes normal-abnormal classification error. We show experimentally that our approach accurately differentiates between normal and abnormal pancreatic tissue. This is, to our knowledge, the first study to use ex-vivo unfixed human pancreatic tissue combined with feature-learning from raw Müller matrix readings for this purpose.

## Introduction

Polarimetry is a powerful technique for analyzing the optical properties of materials and tissues^1^. It involves measuring the polarization state of light, defined by the spatial orientation of the electric field vector with respect to a reference plane. By analyzing the polarization state of a wave of light transmitted through or reflected by a sample, it is possible to gain insight into the underlying structural and functional properties of the sample^2, 3^. Polarimetry has been used in various fields, including biology, material science, and engineering, to study the optical properties of a wide range of materials and tissues^4, 5^.

The use of Müller matrix polarimetry (MMP) for the diagnosis of different types of tissue has been the subject of other studies in recent years^6, 7^. MMP has been shown to be an informative tool for breast tissue characterization^8, 9^, as well as for the differentiation of skin lesions, such as melanoma and non-melanoma skin cancers^10–12^. It has also highlighted differences between normal and diseased ocular tissues, including the cornea and conjunctiva^13, 14^. In addition, MMP has been employed to diagnose gastrointestinal disorders, such as inflammatory bowel disease and colorectal cancer^10, 15–19^ as well as cervical cancer^20–22^, prostate cancer^23, 24^ and giant cell tumor of bone^25^. Commonly, these studies use features derived from the decompositions of the Müller matrix (e.g., diattenuation, retardance, depolarisation, fast axis orientation) since they provide a more physically interpretable reading^26, 27^. Nevertheless, new studies use machine learning algorithms to generate new features tailored to the data and task at hand^28^. These studies demonstrate the versatility and potential of MMP for clinical diagnosis.

However, investigating pancreatic tissue using polarimetry has been limited, particularly for fresh specimens. This is partially due to pancreatic tissue being highly heterogeneous and exhibiting complex optical properties challenging to characterize^29, 30^, combined with the challenging location of the pancreas within the body. Although there are developments in the indirect measurements of polarimetric features via Jones matrix tomography^31^, direct imaging and sample collection are only achievable by complex surgeries^32^. Finally, the technique generates large amounts of data and can be challenging to process and interpret efficiently. However, pancreatic diseases, such as pancreatitis and pancreatic cancer, are major health problems with high morbidity and mortality rates if not diagnosed and treated early^33^. Accurate and reliable methods for diagnosing these diseases are critical for improving patient outcomes.

Driven by the need and despite these challenges, we investigate the capability of MMP to be a valuable tool for diagnosing pancreatic diseases. In this study, we demonstrate the potential of multi-spectral MMP to characterize pancreatic tissue by analyzing fresh specimens with a custom-built device. To overcome the large amounts of data generated, we employ machine learning to process this data and reliably infer corresponding tissue types. Particularly, we measured the Müller matrices of ex-vivo unfixed human pancreatic tissue and used its raw values, leveraging the feature-learning capabilities of the machine-learning model, to derive optimized data representations that minimize normal-abnormal classification error. We show experimentally that our approach accurately differentiates between normal and abnormal pancreatic tissue. To the best of our knowledge, this is the first study to use ex-vivo unfixed human pancreatic tissue combined with a feature-learning algorithm for this purpose.

## Methods

### Data collection

We collected 15 MMP images of pancreatic biopsy specimens from 11 patients undergoing surgery at the Department of visceral surgery and medicine, Bern University Hospital, Inselspital (see Table 1) and handled by the Institute of Tissue Medicine and Pathology, University of Bern. Specimen tissue ranged between $13.3\,mm^{2}$ and $192.7\,mm^{2}$ in size. Informed consent was obtained from all the patients, and all personal data was fully anonymized. This study was approved by the cantonal ethics committee of Bern (KEK BE 2020-00498) and is in line with the declaration of Helsinki.

**Table 1:** Summary of collected data: 11 unique patients, 5 with both normal and abnormal tissue zones, from which 15 samples were extracted, 2 with regions of both normal and abnormal tissues. Saturated pixels were removed from the study.

Label	Patients	Samples	Region area ($mm^{2}$)	Total pixels	Used pixels$^{*}$
Normal	9	9	310	401,784	339,631
Abnormal	7	8	151	195,611	177,073
Total	11	15	461	597,395	516,704

### Acquisition pipeline and gold standard

**Figure 1:**
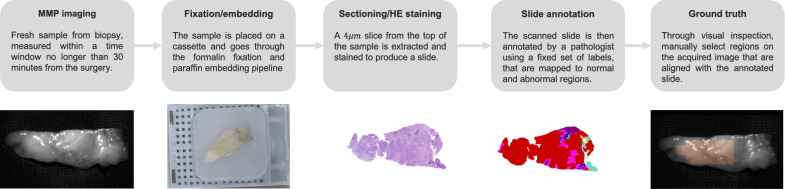
Data acquisition process. Notice that the sample shape in the scanned and annotated slides can be significantly different from the one captured by our MMP device due to the sample passing through the histopathology laboratory pipeline. Following a slide annotation, our data contains both MMP images, and corresponding tissue type classifications at pixel-wise levels.

Biopsies of pancreatic specimens were processed to produce the MMP images with associated annotations to yield a complete dataset. Specifically, we image all tissue specimens using our custom-built MMP device before formalin fixation, paraffin embedding, and sectioning. From HE scanned slides, manual annotations of tissue types were generated and overlayed onto the MMP image. Figure 1 illustrates this pipeline and the following sections describe each stage in detail.

#### MMP imaging

**Figure 2:**
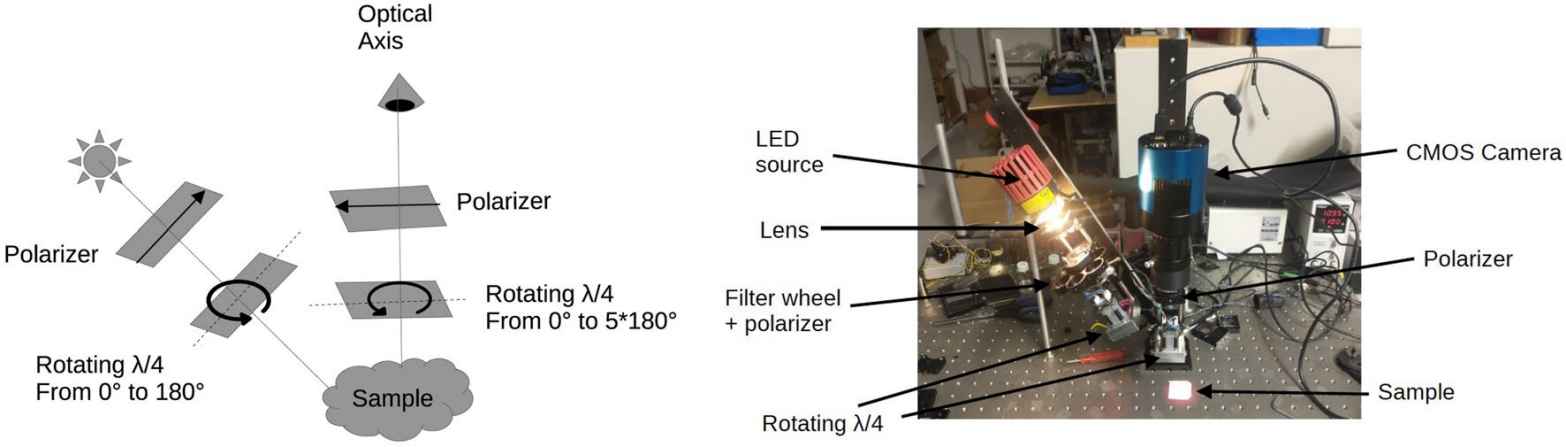
(Left) Schematic representation of our custom-built dual-rotating retarder polarimeter and (Right) visual depiction of our instrument with a phantom sample^35^.

Specimens were first imaged using a custom-built dual-rotating retarder polarimeter^34^ (Fig. 2). The polarimeter consists of a light source, a filter wheel, a polarization state generator, and a polarization state analyzer. We used broadband color filters to restrict the light source to specific wavelengths when passing through the polarization state generator. This generator comprises a fixed linear polarizer and a rotating quarter-wave plate, which generates the polarization state of the wave of light before interacting with the sample. Following interaction with the sample, the outgoing light wave passes through the polarization state analyzer, which comprises a rotating quarter-wave plate followed by a linear polarizer, before being imaged on a CMOS detector. By rotating the wave plates at harmonic frequencies and taking a series of measurements with a fixed color filter, the polarimeter constructs pixel-wise Müller matrices that encode the tissue’s response to any polarization state for the selected wavelength range. This process was repeated for each filter to reconstruct a low-resolution polarisation spectrum.

We imaged specimens using our device at a resolution of 36 pixels/mm, with pixel-wise $4\times {}4$ Müller matrices calculated at the filters’ five central wavelengths (450 nm, 470 nm, 500 nm, 540 nm, and 625 nm) for a total of 80 features per pixel. Müller matrices were scaled by the inverse of their first element, effectively reducing each matrix to 15 degrees of freedom. After normalization, the first elements of the matrices were omitted, thus reducing the number of features per pixel to  75 dimensions. The complete imaging of a sample took roughly 15 minutes on average. To assess the stability of the optical characteristics during these 15 minutes, we took sequential measurements every two minutes from three samples from brain and lung tissue under the same wavelength and exposure time. The results do not show significant changes, indicating measurements in our experimental setup should not be significantly affected by measurement times.

#### Histology and annotation

Following MMP imaging, samples were sent for standard processing in the histopathology laboratory. Among others, formalin fixation, paraffin embedding, cutting, and hematoxylin and eosin (HE) staining were performed, and slides were digitized to high-resolution color images.

A resident pathologist then analyzed all HE images to generate pixel-wise tissue annotations. Specifically, each pixel in the HE images was labeled with a corresponding tissue type of “Normal” (*i.e.*, fat tissue, exocrine pancreas, endocrine pancreas, and stroma) or “Abnormal” (*i.e.*, tumor, desmoplasia, inflammation, fibrosis, and pancreatitis). Annotations were performed using QuPath^36^.

#### Registration to MMP images

To generate the corresponding tissue annotations for the MMP images, the HE annotations are registered to the corresponding MMP images. However, due to histology process causing moderate deformations in the specimen, a registration step is necessary to put pixels between the HE and the MMP images in correspondence. To do this, correspondences were established manually through visual inspection, comparing the geometry, length, and appearance of the structures in the HE image, HE annotations, and the MMP image. Due to this manual and approximate registration process, only pixels in areas with uniform annotations were labeled with high confidence, leaving the remaining pixels unlabeled. By using this registration, HE annotations were then superimposed onto respective MMP images. Table 1 summarizes the complete dataset in terms of pixels annotated.

### Classification of abnormal tissue

We propose to automate the identification of abnormal tissue by establishing a machine learning classification task. Specifically, a multi-layer perceptron^37^ (MLP) was used to classify each pixel of an MMP image as either normal or abnormal. The MLP architecture consisted of a single hidden layer of 64 units and utilized the rectified linear unit (ReLU) activation function.

Using this MLP architecture, we trained two versions of this classifier. The first classifier, **MLP-pol**, was trained using complete multispectral MMP information, with input vectors consisting of 75 measurements of normalized Müller matrices at five different wavelengths for each pixel. The second classifier, **MLP-no-pol**, did not include polarization information in the input, resulting in only 5-dimensional input vectors of multispectral data acquired at each of the five wavelengths. Comparing the performance of the classifiers served to evaluate the importance of polarization information in identifying abnormal tissue.

Both classifiers were trained to predict the abnormal tissue’s probability at the given pixel. Training minimized the standard cross-entropy loss between the predicted probability and the ground truth over the annotated pixels of the training set. This minimization was performed for ten epochs. The training of each model was performed using the Adam optimizer with a learning rate of $10^{-5}$.

### Evaluation

Classifiers were evaluated using k-fold cross-validation, with the data split into $k=4$ folds on a patient-by-patient basis to avoid specimens from the same patient appearing simultaneously in both the training and test splits. For each fold, the models were trained using the training split and evaluated using the test split. The receiver operating characteristic (ROC) curve and the area under the ROC curve (AUC) were reported as metrics to assess the performance of the models. Similarly, we compute model performances when evaluating the entire sample (*i.e.*, specimen classification) whereby considering the specimen abnormal if any pixel in the annotation is abnormal and normal otherwise. A percentile of the prediction score represents the prediction of that sample. We tested percentiles ranging from the 50th up to the 99th. We also report type I and II errors by setting a threshold for the predicted probabilities and identifying the false positives and false negatives.

## Results

**Figure 3:**
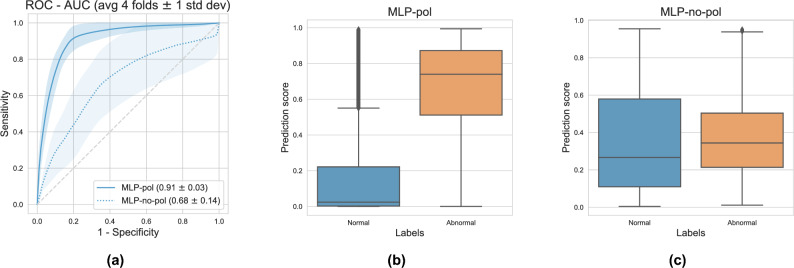
(**a**) ROC curves and AUCs attained on the test set by each evaluated model. Values are averaged over 4 folds and error bars indicate 1 standard deviation. (**b**) Boxplot of prediction probabilities using MLP-pol and (**c**) MLP-no-pol. The prediction scores observed on the MLP-no-pol (**c**) depict a considerable overlap among classes, while a clear class separation is observed on MLP-pol (**b**).

**Figure 4:**
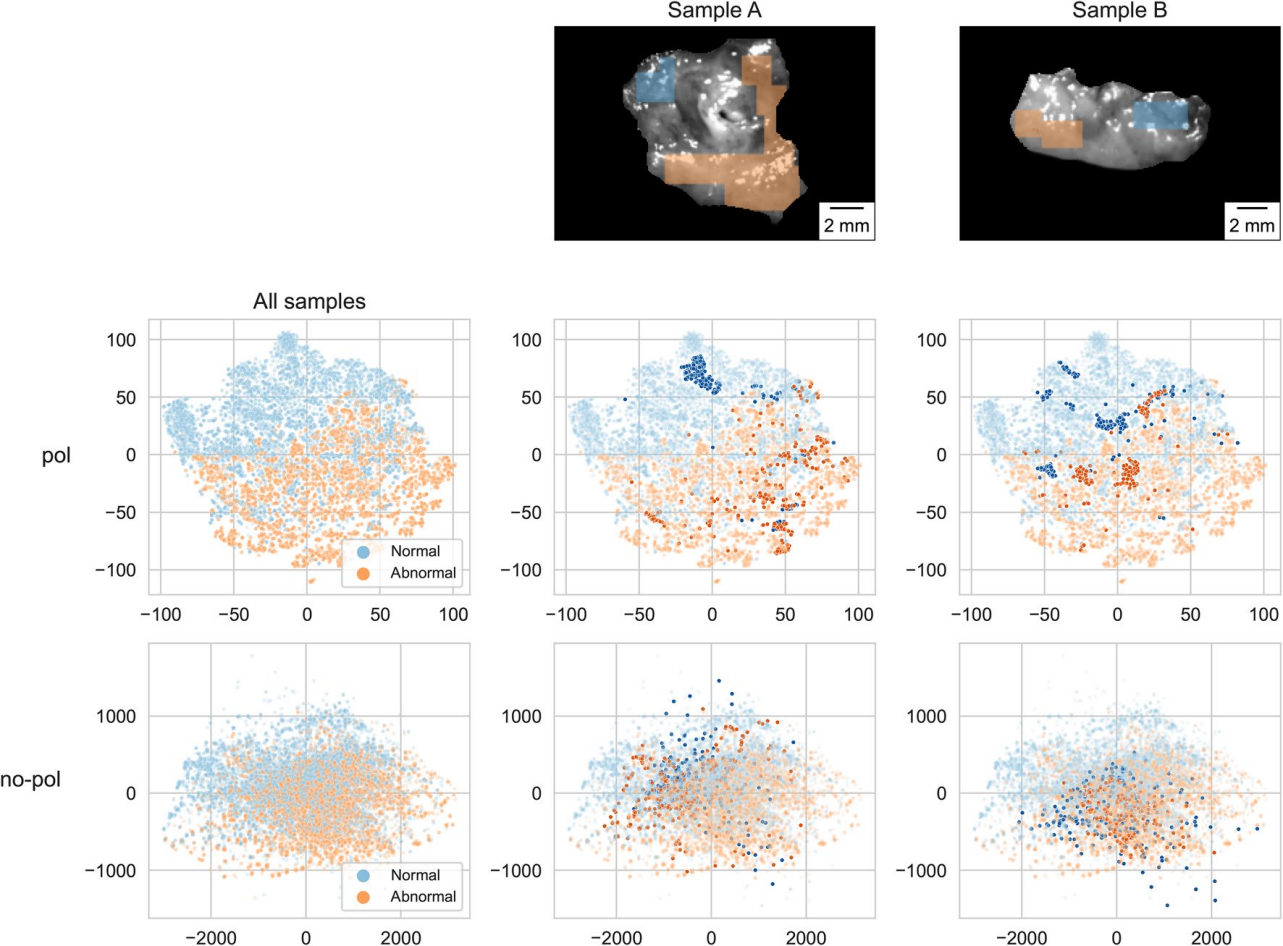
Visual representation of MMP pixels using t-SNE analysis. (First column) t-SNE plots generated using all acquired samples. (Second and third columns) Highlighted observations from two samples containing both tissue types. The pol data generated a t-SNE embedding space where even pixels coming from the same sample are grouped according to their tissue type. The no-pol data was unable to generate an embedding space with clear tissue type separation.

The MLP-pol showed excellent performance in detecting abnormal tissue. Considering abnormal as our positive class, MLP-pol reached an AUC above $90\%$ and sensitivity (true positive rate) and specificity (true negative rate) above $80\%$ (Fig. 3a). Conversely, the performance was significantly worse using MLP-no-pol, with a decrease of more than 15 percentage points in AUC. The use of polarimetry information also increased the classifier’s confidence and reduced the predicted probabilities’ entropy, leading to improved class separability, as shown in Fig. 3b and c. This improved separability was further observed when the input features were visualized in a 2-dimensional space using t-SNE^38^ (see Fig. 4). The use of polarimetry data resulted in distinct clusters of normal and abnormal pixels that were apparent in the input feature space without needing a classifier. On the other hand, no clusters were observed when using multispectral features without polarimetry information. Furthermore, the prediction models, when applied to classify an entire specimen, yielded an AUC of 0.96 and 0.73 MLP-pol and MLP-no-pol, respectively.

**Figure 5:**
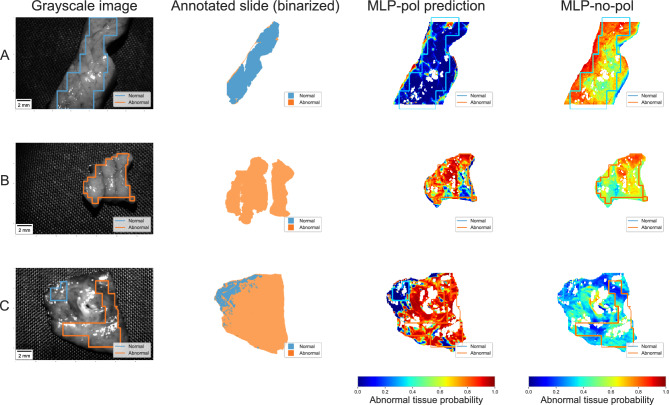
Predictions results for three test samples: (**A**) entirely normal, (**B**) entirely abnormal, and (**C**) with both regions. The probability maps generated by MLP-pol correctly predicted the different tissue types with high confidence, including the two regions within the same sample. Predictions outside the annotated region matched the annotated slide. In contrast, the predictions of MLP-no-pol for samples A and B yielded scores with very low differentiation. On sample C, although it identified differences within the sample, it lacked the confidence presented by MLP-pol.

The qualitative results of both models are shown in Fig. 5. The MLP-no-pol (column 3) appears unable to distinguish between normal and abnormal regions. This is particularly pronounced in samples A and B, where regions of both tissue types were classified with similar prediction scores, confirming the overlap observed in Fig. 3c. While the performance was slightly better in sample C, we still observed low confidence in the separation of both types of tissues, and the prediction seems to follow the visual differences in pixel intensities. In contrast, the MLP-pol classifier does not exhibit these inconsistencies and correctly identifies different tissue types with high confidence. Its predictions also appear accurate outside of the MMP annotated regions, as evidenced by the similarity between the probability maps and the binarized annotated HE slides from the pathologist (column 2).

**Figure 6:**
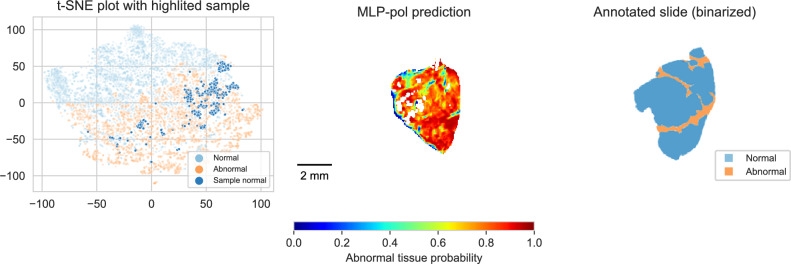
Sample exhibiting a high rate of false positives. t-SNE plot highlighting normal pixels that are located within the abnormal region.

**Figure 7:**
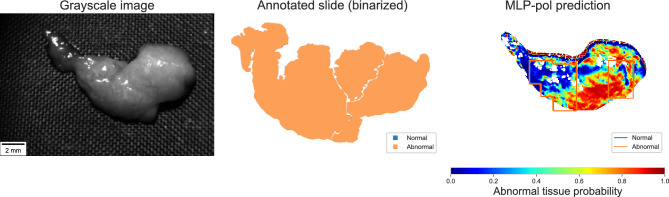
Example of performance errors due to registration misalignment which induces a large false negative ratio. (Left) gray-scale image from our instrument, (Center) binarized slide annotation, and (Right) MLP-pol prediction with registered annotation overlayed.

A probability threshold of 0.43 resulted in an average sensitivity of $80\%$ in the MLP-pol method, with an average of 26% false positives per sample. This large false positive rate was primarily caused by a single specimen in which $90\%$ of its normal pixels were wrongly predicted as abnormal. The majority of pixels of this specimen were located in the unhealthy region of the t-SNE plot as illustrated in Fig. 6). On average, abnormal samples exhibited $19\%$ false negatives per sample, again primarily driven by a single sample with $51\%$ false negatives (see Fig. 7).

## Discussion

The results of our experimental study demonstrate the utility of polarimetry in identifying distinct tissue types within pancreatic samples. We evaluated the performance of a machine learning model utilizing polarimetric information acquired at five distinct wavelengths in differentiating normal and abnormal fresh pancreatic tissue biopsies. Our findings confirm that incorporating polarimetric information results in a significant improvement in classification accuracy compared to the use of multi-wavelength information alone. These results further highlight the challenges faced by surgeons in visually distinguishing between different tissue types in the surgical environment. In contrast, using polarimetry-based methods, such as the one employed in this study, demonstrates a powerful capability for characterizing and differentiating tissue types. Visual inspection of the results supported the quantitative findings and enabled the direct projection of predictions at the pixel level on the polarimetric images.

t-SNE plots for all samples (see Fig. 4) depict embedding spaces generated by the MMP that formed two groups based on their tissue type, while using no-pol features did not generate a clear grouping. Evaluating the highlighted pixels from samples A and B revealed that the pixels from the same sample but with different tissue types are still located correctly within their tissue type group. This supports our claim that the MMP is sensitive enough to overcome some intra-sample correlation and yet be representative of tissue characteristics.

Two samples yielded important inconsistent results. One sample, which appeared normal, had many pixels classified as abnormal. As depicted in the t-SNE plot below (Fig. 6), this sample’s features were found to be closer to the abnormal group than the normal group. One possible explanation for this discrepancy is that it could result from the limited size of the data set, where the feature values presented by this patient fall outside the distribution of the normal patients currently represented in the data set. The second sample yielded a high level of false negatives. Comparing the gray-scale image with the binary ground truth (see Fig. 7) suggests the possibility of registration misalignment, mainly since a significant portion of the sample was accurately classified as abnormal.

**Figure 8:**
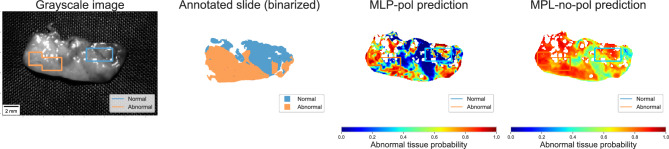
Sample where both prediction models indicated similar regions. The contrast of prediction score among the regions given by MLP-pol is significantly higher, leading to a superior class separation when compared the MLP-no-pol.

From the t-SNE plot in Fig. 4, we could infer that using polarimetric features would yield better classification performances. Nevertheless, there were samples where the MLP-no-pol yielded results closer to the MLP-pol. In Fig. 8, we see that both models were able to identify important regions - the main difference is in the confidence of these predictions. The contrast in prediction scores produced by MLP-pol is clear and consistent with the results on other specimens and across test folds. On the other hand, the scores predicted by MLP-no-pol are in a narrow range that can be found in both normal and abnormal tissues and correlates strongly with differences visible on the grayscale image. This lack of separation among tissue types and consistency negatively impacts its AUC and increases the performance variance across folds, rendering the pixel intensities model unreliable for this task.

The results presented thus far are at a pixel level. However, a proxy for sample-level performance can be obtained by utilizing a percentile of the prediction score for a given sample as the prediction for the entire sample. On the same patient test folds evaluated for the pixel-level model, by starting at the 60th percentile and proceeding up to the 99th percentile, the MPL-pol exhibits an AUC of $96\pm 6$. In contrast, MLP-no-pol only yields an AUC of $73 \pm 18$ at best. The difference in the mean and standard deviation of this sample-level AUC further highlights the consistency of polarimetric information for tissue type differentiation.

There are, however, several limitations in the current study. One of them is that our current instrument design requires one measurement per color filter, and the pixel-wise Müller Matrix calculation algorithm is time-consuming. Each measurement takes approximately 3 minutes, resulting in a total acquisition time of 15 minutes. This is compounded by an existing additional up to 20-minute transport time of samples between the operating room and the imaging system. Consequently, as the tissue samples are analyzed, dehydration occurs and implies that the properties of the samples change over time. While these changes only moderately altered the optical properties of tissue in which we tested the reading’s stability (brain and lung), the metabolic aspect may be more severely impacted. Given that the exact physical process of how tissue sample and polarimetry interact remains unknown, it is likely that our assumption that Müller matrices of tissue are constant over time requires further investigation, in our case, specifically for pancreatic tissue.

Another important limitation of the present study is that after collection, the samples undergo standard histopathological processing, which can alter the geometry of the sample. This renders the registration between HE and our device’s images a challenging task. As a result, misalignment between some samples and their corresponding HE slides can lead to annotation errors. While we have mitigated this by only using annotations from continuous regions, some annotations may be incorrect. Similarly, while the dataset collected and presented here includes over 500’000 MMP data points, these stem from 11 unique patients. As such, the generalization of our results to a broader population should be cautioned.

In summary, however, our results show encouraging evidence that spectro-polarimetry can be an effective differentiation tool for pancreatic tissue subtypes. In the future, we will investigate the ability to differentiate within the abnormal tissues - mainly separating the malignant tumor and desmoplastic reaction from fibrosis or chronic pancreatitis. This could open the door to one more source of information to improve diagnostic accuracy.

## Data Availability

The datasets generated and/or analysed during the current study are not publicly available as they are undergoing intellectual property protection at present, but are available subject to terms and conditions from the corresponding author on reasonable request.
